# Safety, Tolerability, and Metabolic Effects of Long-Acting Cabotegravir and Rilpivirine in HIV Care: A Comprehensive Review

**DOI:** 10.3390/v17081108

**Published:** 2025-08-12

**Authors:** Martina Bottanelli, Antonella Castagna, Camilla Muccini

**Affiliations:** 1 Infectious Diseases Unit, “Alessandro Manzoni” Hospital, ASST-Lecco, 23900 Lecco, Italy; 2 School of Medicine, Vita-Salute San Raffaele University, 20132 Milan, Italy; castagna.antonella@hsr.it; 3 Infectious Diseases Unit, IRCCS San Raffaele Scientific Institute, 20127 Milan, Italy; muccini.camilla@hsr.it

**Keywords:** long-acting, cabotegravir, rilpivirine, antiretroviral therapy, people with HIV, safety, tolerability, side effects

## Abstract

The use of long-acting cabotegravir and rilpivirine (LA CAB/RPV) is a novel approach to manage human immunodeficiency virus (HIV). This injectable regimen offers benefits such as an improved quality of life, reduced stigma and enhanced treatment satisfaction by minimising the need for daily medication adherence. This review summarises the findings of clinical trials and real-world studies on the safety, tolerability and metabolic effects of LA CAB/RPV, which are areas that have received less extensive coverage in previous reviews. Clinical trial data suggest that LA CAB/RPV is generally safe and well tolerated. The most common side effects were injection site reactions, affecting 70–97% of participants. However, these were typically mild and short lived, rarely leading to treatment discontinuation in fewer than 2–3% of cases. Systemic side effects were minimal and comparable to those observed with traditional oral antiretroviral therapy. Real-world studies corroborated these findings, reporting low discontinuation rates due to adverse events. Regarding metabolic impact, clinical trials showed minimal weight gain (an average increase of 1–2 kg over 48–96 weeks) with no significant differences or impact on lipid and glucose levels. Although real-world data are still emerging, they suggest similar trends, including a possible improvement in lipid profiles. Overall, LA CAB/RPV appears to be a safe, well-tolerated and effective treatment option, although longer-term follow-up is needed.

## 1. Introduction

**Figure 1:**
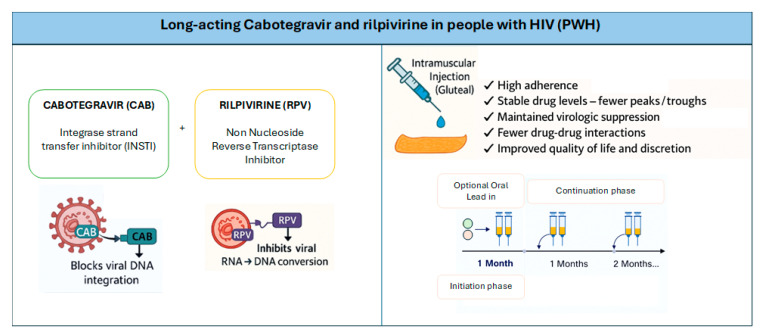
LA CAB/RPV function and mechanisms.

Long-acting cabotegravir and rilpivirine (LA CAB/RPV) represents a paradigm shift in HIV care. Not only does it simplify treatment, it also enhances the quality of life (QoL) of people with HIV (PWH) by reducing adherence barriers, the burden of stigma, and improving overall treatment satisfaction [1,2,3,4]. LA CAB/RPV is the first injectable antiretroviral therapy (ART) regimen recommended by treatment guidelines for maintaining virologic suppression in PWH on stable ART. It was approved for medical use by the European Medicines Agency (EMA) in December 2020 and by the US Food and Drug Administration (FDA) in January 2021 [2]. LA CAB/RPV function and mechanisms are reported in Figure 1.

Several pivotal clinical trials have demonstrated the effectiveness of LA CAB/RPV in maintaining virological suppression, showing non-inferiority to daily oral ART [5,6,7,8,9,10,11,12,13,14,15,16].

In recent years, various studies have provided real-world data on the use of LA CAB/RPV for HIV treatment. These studies have shown significant data reinforcing the findings of clinical trials in terms of effectiveness, safety and patient satisfaction in broad populations in real-life settings [1,17,18,19,20,21,22,23,24,25,26,27,28,29].

To date, a comprehensive review combining data from clinical trials and real-world studies concerning the safety, tolerability and metabolic impact of LA CAB/RPV in people living with HIV (PWH) is lacking in the literature. Recently, two systematic reviews and meta-analyses by Manalu et al. [30] and Wang et al. [31] highlighted the high rate of virological suppression and favourable tolerability profile of LA CAB/RPV in both prevention and treatment settings. However, their research has primarily focused on clinical efficacy endpoints and trial-based safety outcomes without addressing metabolic effects (e.g., changes in weight and lipids) or integrating data from real-world studies, which are becoming increasingly relevant for implementation in routine clinical practice. Our review aims to address this by providing a more detailed analysis of tolerability and metabolic outcomes, particularly focusing on emerging evidence from observational cohorts and real-life settings. To our knowledge, this is one of the first comprehensive reviews to integrate both clinical trial data and real-world evidence in order to evaluate the safety, tolerability, and metabolic impact of LA CAB/RPV in people living with HIV (PWH) in detail. Specifically, we provide an overview of the frequency of adverse events (AEs), including drug-related AEs, serious AEs (SAEs), injection site reactions (ISRs), withdrawals due to AEs and mortality rates associated with LA CAB/RPV therapy. This evaluation has been conducted across both clinical trial data and real-world settings to provide a comprehensive understanding of the safety profile of this novel therapeutic approach. Furthermore, by collecting evidence from both clinical trials and real-world studies, this review explores the potential impact of LA CAB/RPV on weight management and metabolic profiles in PWH, providing valuable insights for clinicians, researchers, and other healthcare professionals involved in HIV care.

## 2. Materials and Methods

A comprehensive literature review was conducted using the PubMed database and the ClinicalTrials.gov clinical trial registry to identify relevant studies. Search terms were selected to cover a broad yet focused scope, ensuring alignment with the review’s objectives. Keywords such as “HIV”, “AIDS”, “long-acting formulations”, “cabotegravir”, “rilpivirine”, “safety” and “tolerability” were used.

Studies providing original data on LA CAB/RPV therapy were included in the review, particularly those examining safety, tolerability and clinical outcomes in diverse populations. Studies with clearly reported methodologies and adequate sample sizes were prioritised to ensure data quality. No additional formal inclusion criteria were applied beyond those described.

Studies were excluded if they were review articles, had incomplete or duplicate data, or were not published in English or lacked a full-text version. This approach ensured the reliability and integrity of the findings.

To ensure the comprehensive inclusion of relevant studies, no predefined publication date range was applied in the literature search. Nevertheless, all the references selected for inclusion were published between September 2017 and July 2025.

The review process involved two distinct stages. First, the titles and abstracts of retrieved articles were evaluated and irrelevant studies were excluded. Secondly, full-text reviews were conducted on potentially relevant articles to confirm their eligibility.

Key data points were extracted from the selected studies, including study design, sample size and safety and tolerability outcomes.

Particular attention was paid to documenting AEs, ISRs, metabolic changes, and weight-related outcomes. The collected data were analysed to provide valuable insights into the impact of LA CAB/RPV therapy in clinical practice.

## 3. Results

### 3.1. Safety and Tolerability of LA CAB/RPV in PWH

The efficacy, safety and tolerability of LA CAB/RPV, when administered as an injectable formulation, have been extensively studied in clinical trials and real-world settings [5,6,7,8,9,10,11,12,13,14,15,16,17,18,19,20,21,22,23,24,25,26,27].

Possible AEs associated with LA CAB/RPV administration have been reported in the EMA and FDA-approved labelled drug data sheets based on the findings of pivotal clinical trials [32,33,34]. Most AEs, particularly ISRs, occur within the first three days after LA CAB/RPV administration and typically resolve within three to seven days. These reactions are most frequent during the initial injections and tend to decrease in both frequency and severity over time, as observed in the FLAIR, ATLAS and ATLAS-2M trials [5,6,7]. SAEs, when reported, generally occur early in treatment and are rarely persistent or severe.

- Injection Site Reactions

The most frequently reported AEs are ISRs, such as pain, swelling, erythema, induration and nodules. These symptoms are usually mild to moderate in severity, transient (typically resolving within three to seven days after injection) and tend to decrease over time without requiring discontinuation of the therapeutic regimen. Despite these common symptoms, many PWH have reported that the benefit of avoiding daily oral ART far outweighs the discomfort associated with injections [32,33,34]. Less commonly, systemic post-injection reactions have been reported, including dyspnoea, agitation, abdominal cramping, musculoskeletal pain, sweating, and changes in blood pressure (vasovagal or presyncopal reactions), which typically subside within a few minutes of the injection [32,33,34].

- Systemic symptoms

Systemic AEs such as fever, fatigue, myalgia, headache, rash, and gastrointestinal symptoms (nausea, diarrhoea) have been widely documented with a lower frequency than ISRs and with rates comparable to oral ART [32,33,34].

- Neuropsychiatric symptoms

Neuropsychiatric symptoms (e.g., depression, anxiety) are rare and substantially comparable to those observed with integrase strand transfer inhibitors. Other neurological AEs occasionally reported are dizziness, vertigo, and insomnia [32,33,34].

- Laboratory abnormalities

Laboratory abnormalities with a worsening grade from baseline have been occasionally reported, especially transaminase elevation and hyperbilirubinemia [32,33,34].

- Serious AEs

SAEs are infrequent, mostly represented by hypersensitivity reactions, including fever, rash, serious liver abnormalities, facial oedema and angioedema. Occasionally, SAEs such as severe skin reactions (i.e., Stevens–Johnson syndrome) and drug reaction with eosinophilia and systemic symptoms (DRESSs) have been documented [32,33,34].

#### 3.1.1. Safety and Tolerability: Data from Clinical Trials

Significant data on the safety and tolerability of LA CAB/RPV were reported in the 48-week results of the first two international, multicentre, open-label phase 3 trials (FLAIR and ATLAS). Further information has since been documented by other trials, essentially reinforcing the results of the initial two pivotal studies. Overall, clinical trials have demonstrated that the LA CAB/RPV regimen is generally safe and well tolerated in PWH, particularly with regard to the low incidence of SAEs, predominantly mild-to-moderate ISRs, minimal clinically significant laboratory abnormalities, and low rates of treatment discontinuation due to AEs. Patient-reported outcomes also indicate high levels of satisfaction and adherence to the injectable regimen compared to daily oral therapy [5,6,7,8,9,10,11,12,13,14,15,16,17,18].

Analyses of the aforementioned studies were primarily based on investigating the frequency of AEs and AE-related withdrawals. Studies comparing the safety of LA CAB/RPV with the standard of care (SOC) for oral ART showed that frequencies of the most common AEs (excluding ISRs) were similar in both groups with substantially overlapping safety profiles [5,6,10,11,12,13,14,15,17]. The most common AEs of LA CAB/RPV, based on data from the ATLAS, ATLAS-2M, FLAIR, LATTE-2, POLAR and SOLAR studies, are reported in Table 1 [5,6,7,8,9,10,11,12,13,14,15,16].

ISRs have by far been the most frequently reported AEs related to LA CAB/RPV administration. Symptoms such as pain, swelling, erythema, induration and nodules at the injection site were frequently reported across all clinical trials. However, they were typically mild to moderate and tended to decline in frequency and severity with continued treatment. Consequently, they were poorly associated with treatment withdrawal.

In the ATLAS and FLAIR trials, up to 84% of participants reported ISRs, but only 1% discontinued treatment due to these reactions [5,6,10]. In the ATLAS-2M study, ISRs led to discontinuation in 2% of PWH receiving Q8W LA CAB/RPV injections and 3% of those receiving Q4W LA CAB/RPV injections [7,8,9]. Similar data have also been reported in the LATTE-2, POLAR and SOLAR studies [13,14,15,16]. While the frequency of LA CAB/RPV withdrawal due to ISRs was generally low, ISRs represented the leading cause of treatment withdrawal among all AEs reported in clinical trials [5,6,7,8,9,10,11,12,13,14,15,16].

In the LA CAB/RPV pivotal clinical trials, a range of parameters was assessed to provide a comprehensive understanding of the safety profile. These evaluations primarily focused on the frequency of any AE, including both general and drug-related AEs, the occurrence of SAEs, and the prevalence and severity of ISRs, which are particularly relevant for injectable regimens. Additionally, the trials examined rates of therapy discontinuation due to AEs, as well as mortality rates, to ensure a thorough assessment of short- and long-term safety outcomes. The data summarised in Table 2 highlight the importance of understanding the clinical efficacy of LA CAB/RPV and its impact on patient tolerance of treatment over time.

The ATLAS trial [5,6] included 616 PWH who were virologically suppressed on SOC oral ART with 308 randomised to the LA CAB/RPV Q4W arm and 308 to the continuation SOC oral ART arm. At week 48, ISRs were the most frequently reported AEs (affecting 83% of patients on the LA CAB/RPV arm), but only 4% were considered severe with only 2% leading to treatment withdrawal. Systemic AEs included headache, fatigue, fever, musculoskeletal pain, nausea and dizziness. Most of these were graded as mild or moderate and rarely led to treatment withdrawal. SAEs occurred in 4% of PWH in the LA CAB/RPV group with a similar rate observed in the SOC oral ART group. Treatment withdrawals due to AEs (including ISRs and systemic AEs) were rare: at 5% in the LA CAB/RPV group. Overall, over 90% of people in the LA CAB/RPV arm expressed satisfaction with the current regimen despite ISRs.

Another pivotal clinical trial was the FLAIR study [6,10,11,12], which assessed the switch from daily oral ART to LA CAB/RPV in treatment-naïve PWH achieving viral suppression (566 PWH randomized, 283 in the LA CAB/RPV arm and 283 in the SOC oral ART arm). At week 48 [10] (and subsequently at weeks 96 [11] and 124 [12]), ISRs were the most common AEs in the LA CAB/RPV arm, mostly classified as mild to moderate in severity, and decreased in incidence over time. Overall, 2% of the 232 PWH in the extension switch population and 2% of the 283 PWH in the randomly assigned LA CAB/RPV group withdrew due to ISRs. Other AEs leading to withdrawal were infrequent, occurring in only 1% of people in the extension switch population (one in the direct-to-injection group and two in the oral lead-in group) after 24 weeks of LA CAB/RPV and in 5% of people in the randomly assigned LA CAB/RPV group up to week 124. No deaths occurred in the extension phase. Results from week 124 for PWH who were originally assigned to LA CAB/RPV showed durable maintenance therapy with a favourable safety profile.

The ATLAS-2M study [7,8,9] evaluated the efficacy and safety of extending the LA CAB/RPV dosing interval from Q4W to Q8W. The study aimed to determine whether a less frequent dosing regimen would provide similar therapeutic outcomes while maintaining the safety and tolerability profile observed with Q4W dosing. At week 96, both the Q8W and Q4W LA CAB/RPV regimens were found to be equally effective in maintaining virological suppression in PWH who were previously stable on ART. In terms of safety and tolerability, the two regimens had similar profiles. The most commonly reported AEs were ISRs, which were generally mild to moderate, transient and resolved without the need for significant intervention. There were no significant differences in the rates of other AEs between the two groups, suggesting that extending the dosing interval to Q8W did not increase the risk of systemic AEs or SAEs compared to the Q4W regimen. The comparable safety and tolerability profiles of the Q8W and Q4W regimens, coupled with the treatment’s efficacy, strongly support the use of the Q8W dosing regimen as a viable therapeutic option. This could improve adherence and reduce the burden of frequent clinic visits while maintaining the high level of efficacy and safety expected from this treatment combination.

Further research was conducted with LATTE-2 phase 2b trial [14,15], which compared the efficacy, safety and tolerability of LA CAB/RPV Q4W and LA CAB/RPV Q8W. The results at week 96 indicated a consistent safety profile with that observed in other studies, supporting the feasibility of less frequent dosing of LA CAB/RPV. The overall safety profile at week 256 of the LATTE-2 study was consistent with the data observed at week 96 of the LATTE-2 study and in the phase 3 ATLAS, FLAIR and ATLAS-2M studies. Although ISRs were frequent, most were mild/moderate, of short duration, and decreased over time. Over approximately five years of LA CAB/RPV treatment, four (2%) PWH from the randomised Q8W/Q4W groups withdrew due to ISRs, two of whom discontinued treatment after 24 weeks or less. After approximately three years of LA CAB/RPV in the extension switch groups, one (2%) person living with HIV withdrew due to an ISR after 60 weeks. By week 256, three (3%) and 20 (17%) PWH) in the randomised Q8W and Q4W groups, respectively, had experienced AEs leading to withdrawal. Of these, two (2%) and eight (7%) participants, respectively, reported treatment-related AEs leading to withdrawal. The cumulative proportion of participants who withdrew due to AEs through week 256 was higher in the randomised Q4W group (17%) than in the Q8W group (3%). SAEs occurred in 52 (23%) and 7 (16%) participants in the randomised Q8W and Q4W groups, respectively. The most frequently reported SAEs in the randomised Q8W or Q4W groups were acute kidney injury and suicide attempt with each SAE occurring in two participants in the Q8W group and neither being treatment related.

In the POLAR study [16], 90 PWH received LA CAB/RPV once every eight weeks. At the 52-week follow-up, none of the participants experienced an HIV VL of ≥50 copies/mL or met the VF criteria. In terms of safety, LA CAB/RPV demonstrated a favourable profile. ISRs were reported, but they were generally transient and well tolerated by participants. Systemic AEs, such as headache or fatigue, occurred at rates consistent with those observed in previous studies. Another key study [13] evaluated the safety and tolerability of LA CAB/RPV Q8W compared to the daily oral regimen of bictegravir/emtricitabine/tenofovir alafenamide (BIC/F/TAF). ISRs were reported in a significant proportion of PWH in the LA CAB/RPV arm, but approximately 98% of ISRs were mild to moderate and decreased over time. Furthermore, only 2% of participants discontinued treatment due to ISRs. The overall incidence of systemic AEs was comparable between the two arms with common AEs including headache and diarrhoea. SAEs were infrequent and occurred at similar rates in both groups.

#### 3.1.2. Safety and Tolerability: Data from Real-World Studies

Real-world studies evaluating LA CAB/RPV [18,19,20,21,22,23,24,25,26,27,28] have consistently confirmed the favourable safety and tolerability profiles observed in clinical trials.

ISRs were the most frequently reported AEs across all real-world studies, predominantly mild-to-moderate and transient, decreasing over time. The discontinuation rate was low (approximately 1–2% of PWH on LA CAB/RPV). Systemic AEs such as fatigue, headache, fever and myalgia have also been documented with frequencies similar to those reported in clinical trials. SAEs were generally rare, which was consistent with clinical trial data. No new safety signals have been identified in real-world settings.

The safety and tolerability findings of the main real-world studies on LA CAB/RPV in PWH are reported in Table 3.

In the OPERA study [19,20], the safety and tolerability of LA CAB/RPV were evaluated retrospectively across different US clinics. The study population included all adult PWH who received their first LA CAB/RPV injection between 21 January 2021 and 28 February 2022. ISRs were commonly reported among PWH, but the majority were mild to moderate in intensity and did not lead to treatment withdrawal. Systemic AEs were infrequent, primarily consisting of headaches and fatigue, and no significant SAEs were attributed to LA CAB/RPV treatment. The regimen was generally well tolerated with manageable ISRs and minimal systemic adverse events. These findings support the favourable safety and tolerability profile of LA CAB/RPV in routine clinical practice [20].

Further investigation was conducted, including the BEYOND study [21,22], which was designed to evaluate the outcomes and experiences of 308 PWH initiating LA CAB/RPV in the US. The study confirmed the favourable safety and tolerability profile of LA CAB/RPV in real-life settings. At week 52, AEs were reported in 74/308 (24%) PWH, but only six (2%) reported an SAE, and 13 (4%) reported a drug-related AE (excluding ISRs). ISRs occurred in 30 (10%) PWH. Treatment withdrawals were rare with approximately 2% of PWH discontinuing LA CAB/RPV due to AEs. The most common reasons for discontinuation were medication cost or access issues and individual preference.

Another real-world study, the ongoing nationwide observational SHCS-879 study [23] within the Swiss HIV Cohort Study (SHCS), focused on the real-world implementation of LA CAB/RPV in PWH. The regimen was generally well tolerated, with manageable ISRs and minimal systemic AEs, which is essentially in line with the findings of previous studies.

Building on this, the JABS monocentric study [24] aimed to evaluate the implementation of LA CAB/RPV in a real-world Australian setting. This study included 60 PWH, 60% of whom had one or more identified complexity or vulnerability factors, and 27% of whom had a history of non-adherence to ART. At week 52, LA CAB/RPV was associated with very high adherence to injections (97%) and a favourable safety profile (the ISR rate was 29% and no SAEs occurred).

In the CARLOS study [28], a 3-year, multicentre, non-interventional German cohort study including 236 PWH who switched from suppressive daily oral ART to LA CAB/RPV once every 8 weeks, the tolerability profile of LA CAB/RPV was consistent with observations from clinical trials, with a high frequency of ISRs, but these were always mild-to-moderate in intensity. Six patients (2.7%) discontinued treatment due to ISRs. The vast majority of PWH were adherent to injections in routine clinical practice, and treatment satisfaction increased significantly following the switch from oral ART to LA CAB/RPV Q8W. It should be noted that these data were presented at an international conference but have not yet been published in a peer-reviewed scientific journal.

The SCohoLART study [1] was an Italian, single-centre, prospective cohort study including PWH on virological suppression who switched to bimonthly LA CAB/RPV Q8W. At month 12, of the 514 PWH enrolled, 52 (10.1%) experienced treatment discontinuation, including 4 (0.8%) due to virologic failure (VF). The 12-month cumulative probability of withdrawal was 11% with ISRs being the main cause (28.8%). The one-year cumulative probability of treatment discontinuation with LA CAB/RPV in this cohort of PWH was consistent with the results of other real-life studies. Moreover, the findings of the SCohoLART study were essentially in line with those of clinical trials, demonstrating the efficacy, safety and tolerability of LA CAB/RPV in a population with a higher median age and longer duration of ART exposure.

### 3.2. Weight and Metabolic Changes in PWH Switching to LA CAB/RPV

Despite growing clinical interest, the impact of LA CAB/RPV on weight and metabolic parameters remains an under-explored aspect of HIV care.

Weight gain and metabolic changes, which are often associated with certain ART regimens, can increase the risk of cardiovascular disease (CVD), diabetes, and other metabolic comorbidities. These factors can affect the prognosis, overall health, and quality of life of PWH. A comprehensive review of clinical trials and real-world studies is therefore crucial for understanding the potential impact of switching to LA CAB/RPV on weight, lipid profiles and overall metabolic health.

While the exact mechanisms are unclear, several hypotheses have been put forward to explain potential metabolic changes following a switch to LA CAB/RPV. One possible explanation is the removal of TAF and/or boosting agents (e.g., ritonavir and cobicistat), both of which have been associated with weight gain and unfavourable lipid profiles, as well as the overall simplification of therapy [35,36]. Additionally, the pharmacokinetic profile of long-acting injectable formulations may result in more stable plasma concentrations over time compared to oral ART, which could have a favourable impact on metabolic homeostasis [5,6,14]. Lastly, improvements in treatment satisfaction and reduction in stigma may lead to better psychological well-being and health behaviours, indirectly influencing weight and metabolism [1,28].

#### 3.2.1. Weight and Metabolic Changes: Data from Clinical Trials

Changes in body weight and metabolism were evaluated in the pivotal clinical trials of LA CAB/RPV. The main data are reported in Table 4.

In the ATLAS trial, minimal weight gain (less than 1 kg) was observed among PWH on LA CAB/RPV at week 48; specifically, changes were similar to those observed in the oral ART group prior to the switch. Regarding the metabolic profile, there were no significant changes in total cholesterol (TC) or low-density lipoprotein cholesterol (LDL-c), while high-density lipoprotein cholesterol (HDL-c) levels remained unchanged or showed slight improvement [5].

In the FLAIR trial, modest weight increases were observed in participants after switching to LA CAB/RPV with an average weight gain of approximately 1–2 kg over 96 weeks (weight changes were comparable to those in the oral ART group). No clinically significant changes in lipid parameters were reported. Switching to LA CAB/RPV did not appear to adversely affect TC, triglyceride (TG) levels or glucose metabolism [6,10,11,12].

In the ATLAS-2M study, clinically insignificant weight gains have been documented in PWH on LA CAB/RPV Q4W and Q8W. Moreover, lipid and glucose levels remained consistent across both dosing schedules. No significant differences in metabolic outcomes were observed between the Q4W and Q8W dosing groups [7,8,9].

In the LATTE-2 trial, PWH on the LA CAB/RPV arm experienced modest weight increases at week 96 (approximately +1.5 kg in the Q4W dosing group and approximately +1.3 kg in the Q8W dosing group). These changes were comparable to those observed in the daily oral ART group, suggesting that the injectable formulation has no significant additional impact on weight. Regarding the metabolic profile, TC, LDL-c and HDL-c levels remained stable across all treatment arms, and the TC/HDL-c ratio showed no significant change over the study period. Furthermore, no changes in fasting glucose and insulin levels were detected in either group [14].

Of the various clinical trials, the SOLAR study has focused most on weight change and metabolic aspects. It compared weight, anthropometric, and metabolic changes among 454 PWH who switched to LA CAB/RPV Q8W and 227 PWH who continued BIC/F/TAF in the first year of follow-up. In the LA CAB/RPV group, PWH experienced a median weight loss of −0.4 kg over the study period, whereas in the BIC/F/TAF group, PWH experienced a median weight gain of +0.05 kg, reflecting stable weight with minimal changes. While the difference in weight change between the two groups was not statistically significant, it did suggest a trend towards stabilisation or slight reduction in weight with LA CAB/RPV compared to BIC/F/TAF. Regarding the lipid profile, slight decreases in TC and LDL-c were reported in the LA CAB/RPV group, while HDL-c remained stable throughout the study. Furthermore, fasting glucose and insulin levels remained stable in both the LA CAB/RPV and BIC/F/TAF groups with only minor non-statistically significant differences observed between the groups. Overall, the metabolic impact of LA CAB/RPV appeared substantially neutral, with minimal changes in metabolic parameters, and it was similar in the two groups. There were no differences in the proportions of PWH with metabolic syndrome, abdominal obesity, or insulin resistance [13].

Across all the aforementioned clinical trials, PWH who switched to LA CAB/RPV experienced weight changes that were generally modest, which are defined here as slight increases ranging between 1 and 2 kg over 48 to 96 weeks or negligible changes such as weight stabilisation or minimal loss (as reported in Table 4). Furthermore, there were no significant increases in TC or LDL-c, and no relevant changes in glucose or insulin levels were observed.

#### 3.2.2. Weight and Metabolic Changes: Data from Real-World Studies

To date, there are limited data on weight and metabolic changes after switching to LA CAB/RPV in PWH in real-world settings [18,19,20,21,22,23,24,25,26,27,36,37,38,39,40].

Serris et al. recently conducted a retrospective, single-centre study including 126 PWH who initiated LA CAB/RPV between 2014 and 2022 in a real-world setting. After a median follow-up time of 9 months, no significant weight changes were observed [29].

Further data on weight and metabolic changes in PWH on LA CAB/RPV were published by Damas et al. [37]. They evaluated weight and metabolic changes in PWH who had discontinued ART containing TAF with 6555 participants included in the study. Of these participants, 5485 (83.7%) continued TAF-based ART, while 1070 (16.3%) switched to TAF-free regimens. Discontinuing TAF was associated with an adjusted mean weight loss of 0.54 kg after 12 months. Specifically, switching from TAF to TDF resulted in an adjusted mean weight decrease of −1.84 kg, alongside reductions in TC of −0.44 mmol/L and TG of −0.38 mmol/L. Conversely, switching to DTG/3TC or LA CAB/RPV (in 115 participants) did not lead to significant weight changes after 12 months with a mean reduction of 0.58 kg observed in the LA CAB/RPV group.

Furthermore, Adachi et al. evaluated the impact on inflammatory and lipid profiles of LA CAB/RPV therapy in PWH in their retrospective cohort study [38]. The study included 78 PWH who were stratified into two groups: those who had previously been on TAF-based regimens (42 PWH) and those who had previously been on dolutegravir-based regimens (32 PWH). All participants had been on an oral lead-in with CAB plus RPV for at least one month. Over 8 months, inflammatory biomarkers such as C-reactive protein (CRP) and the CD4/CD8 ratio were measured at multiple timepoints along with lipid profiles including HDL-c, LDL-c and the TC/HDL-c ratio. The study used a longitudinal, within-subjects comparison approach to assess changes over time without including an external control group. The results showed no significant changes in CRP or the CD4/CD8 ratio following the switch to LA CAB/RPV. Regarding lipid profiles, there was a significant increase in HDL-c levels and a decrease in the TC/HDL-c ratio, while LDL-c levels remained unchanged.

Similar data on the impact of LA CAB/RPV on the lipid profile of PWH have been observed in the SCohoLART study [1,36]. In individuals switching to LA CAB/RPV, a statistically significant increase in HDL-c and a concomitant reduction in the TC/HDL-c ratio were observed. Specifically, the mean changes in HDL-C and the TC/HDL-C ratio were +3.0 mg/dL/year (95% CI, 1.9–4.1) and −0.2/year (−0.3 to −0.1), respectively (both *p* < 0.001), while no significant changes in weight or other metabolic parameters were observed.

Longer follow-up and larger datasets are required to validate the potential favourable impact of the LA CAB/RPV regimen on the lipid profile and, consequently, on cardiovascular risk in people with HIV.

Regarding weight and metabolic outcomes, key clinical trials have reported minimal, clinically insignificant weight changes as well as overall stability in metabolic parameters (including lipid profiles and glucose metabolism) after switching to the LA CAB/RPV regimen. However, real-world data on the impact of LA CAB/RPV on weight and metabolic profiles in PWH remain limited.

## 4. Conclusions

Consistent findings from both clinical trials and real-world studies indicate that the LA CAB/RPV regimen generally has a favourable safety and tolerability profile in PWH.

ISRs were the most frequent AEs, typically mild to moderate, transient, and rarely resulting in treatment discontinuation. The rates of systemic AEs with the LA CAB/RPV regimen were similar to those commonly seen with oral ART. No unexpected or severe safety issues emerged during long-term follow-up.

Regarding weight and metabolic outcomes, key clinical trials reported minimal and clinically insignificant weight changes as well as overall stability in metabolic parameters (including lipid profiles and glucose metabolism) after switching to the LA CAB/RPV regimen. However, real-world data on the impact of the LA CAB/RPV regimen on weight and metabolic profiles in PWH remain limited. Nevertheless, the promising results from the SCohoLART study suggest a beneficial effect on the lipid profile and a neutral effect on weight and other metabolic parameters, which is consistent with the findings of the clinical trials.

The limitations of this review should be acknowledged. Firstly, the available real-world evidence on the long-term metabolic and cardiovascular outcomes of LA CAB/RPV in PWH is limited. Secondly, the included studies vary in design, population characteristics, follow-up duration and outcome reporting, which may limit the comparability and generalisability of the findings. Lastly, as with any narrative review, there is a potential for publication bias and selective reporting, which could influence the overall interpretation of the data.

In conclusion, clinical trials and real-world studies have widely documented that LA CAB/RPV is an effective and well-tolerated treatment option for virologically suppressed PWH with minimal impact on weight and metabolism. However, the promising findings regarding the use of LA CAB/RPV are based on studies with relatively short follow-up periods. A notable exception is the LATTE-2 study, which provides longitudinal data extending up to five years and offering valuable insights into the long-term efficacy and safety of this regimen. However, further research is necessary to comprehensively define the role of LA CAB/RPV in personalised HIV care. This includes extended longitudinal studies to provide more robust data on the long-term safety and durability of virological suppression as well as the potential for late-emerging AEs. Longer-term studies will also help to clarify the sustained impact of LA CAB/RPV on metabolic and cardiovascular health, which are critical factors for ageing PWH. Given the heterogeneity of PWH demographics, comorbidities and social determinants of health, future research should prioritise real-world observational studies that include broader and more diverse cohorts. This is particularly important for underrepresented groups, such as adolescents (as in the IMPAACT 2017/MOCHA study [41]), women, older adults and individuals with multiple comorbidities or coinfections. While initial findings suggest a minimal impact of LA CAB/RPV on weight and metabolic parameters, comprehensive metabolic profiling in larger, more diverse populations is required. Future studies should systematically evaluate lipid profiles, insulin resistance, glucose metabolism and cardiovascular risk markers to fully characterise the metabolic safety of LA CAB/RPV particularly over prolonged treatment durations.

As LA CAB/RPV becomes more widely used in clinical practice, it will be important to conduct studies that explore the barriers and facilitators to access, the mechanisms that support adherence, the cost-effectiveness and the integration into health systems. This will ensure the equitable delivery of this promising regimen. Addressing these future research priorities will enable the field to better understand the full potential and limitations of LA CAB/RPV, ultimately optimising personalised HIV care across diverse patient populations and clinical settings.

## Figures and Tables

**Table 1:** Most common AEs of LA CAB/RPV based on data from the ATLAS, ATLAS-2M, FLAIR, LATTE-2, POLAR and SOLAR clinical trials. Abbreviations: ISRs, injection site reactions.

AEs	Frequency (%)	Description
ISRs	70–97%	Pain, swelling, nodules, or erythema. Mostly mild-to-moderate in intensity. Persistent nodules have been observed, generally asymptomatic.
Headache	5–12%	Generally mild.
Pyrexia	5–10%	Occasionally post-injection.
Fatigue	5–8%	Typically transient and mild-to-moderate in intensity.
Myalgia	3–7%	Muscle pain reported in some participants.
Nausea	3–6%	Mild gastrointestinal discomfort.
Dizziness	2–5%	Typically transient.
Rash	<5%	Includes mild allergic reactions; rarely leads to discontinuation.
Diarrhoea	<5%	Mild gastrointestinal discomfort.
Depressive Disorders	<10%	Depression, mood changes.
Insomnia	<10%	Difficulty sleeping reported by some participants.

**Table 2:** Safety and tolerability of LA CAB/RPV in PWH: results of the main clinical trials [expressed in terms of frequency (*n*, %)].

Trial	Treatment Arms	Safety and Tolerability					
		Any AEs	Drug-Related AEs	SAEs	ISRs	AEs-Related Withdrawal	Death
FLAIR (48 w) [6]	Q4W IM	267/283 (94%)	236/283 (83%)	18/283 (6%)	227/283 (80%)	9/283 (3%)	0 (0%)
	PO	225/283 (79%)	28/283 (10%)	12/283 (4%)	-	4/283 (1%)	0 (0%)
FLAIR (96 w) [11]	Q4W IM	274/283 (97%)	246/283 (87%)	24/283 (8%)	245/283 (88%)	14/283 (5%)	0 (0%)
	PO	242/283 (86%)	33/283 (12%)	22/283 (8%))	-	4/283 (1%)	0 (0%)
FLAIR (124 w) [12]	DTI	102/111 (92%)	86/111 (77%)	4/111 (4%)	-	1/111 (1%)	0 (0%)
	OLI	100/121 (83%)	79/121 (65%)	5/121 (4%)	-	2/121 (2%)	0 (0%)
	RA LA arm	276/283 (98%)	248/283 (88%)	33/283 (12%)	-	15/283 (5%)	0 (0%)
ATLAS (48 w) [6]	Q4W IM	294/308 (95%)	255/308 (83%)	13/308 (4%)	250/308 (83%)	14/308 (4%)	0 (0%)
	PO	220/308 (71%)	8/308 (3%)	14/308 (4%)	-	5/308 (2%)	1/308 (0.3%)
ATLAS-2M (96 w) [7,8,9]	Q4W IM	499/523 (95%)	413/523 (79%)	28/523 (5%)	400/517 (77%)	19/523 (4%)	1/523 (0.2%)
	Q8W IM	488/522 (93%)	415/522 (80%)	33/522 (6%)	412/516 (80%)	18/522 (4%)	1/522 (0.2%)
LATTE-2 (96 w) [14]	Q4W IM	-	-	11/115 (10%)	112/115 (97%)	8/115 (7%)	-
	Q8W IM	-	-	11/115 (10%)	110/115 (96%)	2/115 (2%)	-
	PO	-	-	7/56 (13%)	-	1/56 (2%)	-
LATTE-2 (256 w) Extension period [15]	Q4W IM	115/115 (100%)	-	27/115 (23%)	-	20/115 (17%)	3/115 (3%)
	Q8W IM	115/115 (100%)	-	25/115 (22%)	-	3/115 (3%)	0 (0%)
	Q4W OLD-IM	10/10 (100%)	-	1/10 (10%)	-	1/10 (10%)	0 (0%)
	Q8W OLD-IM	34/34 (100%)	-	6/34 (18%)	-	1/34 (3%)	0 (0%)
POLAR (52 w) [16]	Q8W IM	86/90 (96%)	65/90 (7%)	5/90 (6%)	70/90 (78%)	1/90 (1%)	-
	PO	3/7 (43%)	1/7 (14%)	1/7 (14%)	-	0/7 (0%)	-
SOLAR (52 w) [13]	Q8W IM	405/454 (89%)	327/454 (72%)	21/454 (5%)	70%	25/454 (6%)	-
	PO BIC/F/TAF	172/227 (76%)	2/227 (1%)	15/227 (7%)	-	2/227 (1%)	-

Abbreviations: Q4W, every four weeks; Q8W, every eight weeks; PO, per os; DTI, direct to injection; OLI, oral lead in; RA, randomly assigned; IM, intramuscular; OLD, optimised loading dose; BIC/F/TAF, bictegravir/emtricitabine/tenofovir alafenamide fumarate; AEs, adverse events; SAEs, serious AEs; ISRs, injection site reactions.

**Table 3:** Safety and tolerability of LA CAB/RPV in PWH in the main real-world studies.

Study	Country	ISRs	Systemic AEs	SAEs (%)	Discontinuation Rate (%)	AEs-Related Discontinuation Rate (%)
OPERA [19,20]	United States	Common (80–90%), predominantly mild-to-moderate	10–20% PWH (e.g., headache, fever, fatigue)	<2%	13%	-
BEYOND [21,22]	United States	Common (10%), mostly mild-to-moderate	Common	2%	13%	-
SHCS-879 [35]	Swiss	Common (46%), mostly mild-to-moderate	Common (pyrexia 9%, fatigue 10%, headache 3%)	-	-	-
JABS [24]	Australia	Common (29%), mostly mild-to-moderate	-	No SAEs reported	10%	3%
CARLOS [28]	Germany	Common (28%, all mild-to-moderate)	Common systemic AEs were pyrexia (4%) and headache (3%)	0.4%	8%	2.5% (6/236 PWH, mostly due to ISRs)
SCohoLART [1]	Italy	Common	1.5% PWH (pyrexia 0.6%, fatigue 0.4%, 0.4%, joint stiffness 0.4%, arthralgia 0.2%)	0.2%	10%	7% (65% of all discontinuations, of which 28% were ISRs)

Abbreviations: AEs, adverse events; ISRs, injection site reactions; SAEs, serious AEs; PWH, people with HIV.

**Table 4:** Weight and metabolic changes after switching to LA CAB/RPV from the pivotal clinical trials.

Trial	Weight Changes	Lipid Profile Changes	Glucose/Metabolic Changes
ATLAS [6]	Minimal weight gain (<1 kg) over 48 weeks. Comparable to oral ART.	Lipid parameters remained stable, with no significant changes in TC or LDL.	No significant changes in glucose metabolism reported.
FLAIR [6,11,12]	Modest weight gain (1–2 kg) over 96 weeks, similar to oral ART.	No significant changes in TC, LDL, TG or HDL levels.	Glucose metabolism remained stable, with no increased risk of insulin resistance.
ATLAS-2M [9]	Weight gain of ~1–1.5 kg over 48 weeks, comparable across Q4W and Q8W dosing.	Stable lipid profile. Slight improvements in HDL levels in some participants.	No clinically relevant changes in glucose or insulin levels.
LATTE-2 [14,15]	Modest weight gain (~1.3–1.5 kg) over 96 weeks in both Q4W and Q8W groups.	Stable lipid parameters across all treatment groups.	Fasting glucose and insulin levels remained within normal ranges.
SOLAR [13]	Median weight change: −0.4 kg (LA CAB/RPV) vs. +0.05 kg (BIC/FTC/TAF) over 12 months.	Slight decreases in TC and LDL; HDL levels stable.	No adverse effects on glucose metabolism reported.

Abbreviations: ART, antiretroviral therapy; Q4W, every four weeks; Q8W, every eight weeks; LA CAB/RPV, long-acting cabotegravir and rilpivirine; TC, total cholesterol; LDL, low-density lipoprotein cholesterol; HDL, high-density lipoprotein cholesterol; TG, triglycerides; BIC/FTC/TAF, bictegravir/emtricitabine/tenofovir alafenamide.

## Abbreviations

AE	Adverse Event
ART	Antiretroviral Therapy
BIC/F/TAF	Bictegravir/Emtricitabine/Tenofovir Alafenamide
CAB	Cabotegravir
CVD	Cardiovascular Disease
DRESS	Drug Reaction with Eosinophilia and Systemic Symptoms
DTG	Dolutegravir
DTI	Direct-to-Injection
EMA	European Medicines Agency
FDA	Food and Drug Administration
HDL-c	High-Density Lipoprotein Cholesterol
IM	Intramuscular
ISR	Injection Site Reaction
LA CAB/RPV	Long-Acting Cabotegravir and Rilpivirine
LDL-c	Low-Density Lipoprotein Cholesterol
OLD	Optimized Loading Dose
OLI	Oral Lead-In
PO	Per Os (by mouth)
PWH	People With HIV
QoL	Quality of Life
Q4W	Every Four Weeks
Q8W	Every Eight Weeks
RPV	Rilpivirine
SAE	Serious Adverse Event
SOC	Standard of Care
TAF	Tenofovir Alafenamide Fumarate
TC	Total Cholesterol
TDF	Tenofovir Disoproxil Fumarate
TG	Triglycerides
VF	Virological Failure
VL	Viral Load
